# The Gut Microbiome and Individual-Specific Responses to Diet

**DOI:** 10.1128/mSystems.00665-20

**Published:** 2020-09-29

**Authors:** Avner Leshem, Eran Segal, Eran Elinav

**Affiliations:** a Department of Immunology, Weizmann Institute of Science, Rehovot, Israel; b Department of Surgery, Tel Aviv Sourasky Medical Center, Tel Aviv, Israel; c Department of Computer Science and Applied Mathematics, Weizmann Institute of Science, Rehovot, Israel; d Department of Molecular Cell Biology, Weizmann Institute of Science, Rehovot, Israel; e Division of Microbiome and Cancer, DKFZ, Heidelberg, Germany; Institute for Systems Biology

**Keywords:** machine learning, microbiome, personalized nutrition

## Abstract

Nutritional content and timing are increasingly appreciated to constitute important human variables collectively impacting all aspects of human physiology and disease. However, person-specific mechanisms driving nutritional impacts on the human host remain incompletely understood, while current dietary recommendations remain empirical and nonpersonalized. Precision nutrition aims to harness individualized bodies of data, including the human gut microbiome, in predicting person-specific physiological responses (such as glycemic responses) to food. With these advances notwithstanding, many unknowns remain, including the long-term efficacy of such interventions in delaying or reversing human metabolic disease, mechanisms driving these dietary effects, and the extent of the contribution of the gut microbiome to these processes. We summarize these conceptual advances, while highlighting challenges and means of addressing them in the next decade of study of precision medicine, toward generation of insights that may help to evolve precision nutrition as an effective future tool in a variety of “multifactorial” human disorders.

## Abstract (precis)

Nutritional content and timing are increasingly appreciated to constitute important human variables collectively impacting all aspects of human physiology and disease. However, person-specific mechanisms driving nutritional impacts on the human host remain incompletely understood, while current dietary recommendations remain empirical and nonpersonalized. Precision nutrition aims to harness individualized bodies of data, including the human gut microbiome, in predicting person-specific physiological responses (such as glycemic responses) to food.

## NUTRITION-MICROBIOME CROSS TALK

### Food digestion and absorption.

Mammalian digestion is initiated by cognitive food perception, which stimulates the production of oral saliva and gastric secretions (1). Later on, the passage of a food bolus through the esophagus and stomach further stimulates the secretion of biliary and pancreatic secretions that play a fundamental role in food decomposition and digestion. Absorption of dietary nutrients takes place mainly in the small intestine, where structures called villi and microvilli greatly increase the mucosal surface area, thereby enhancing its absorptive capacity. Residues of food that was not absorbed in the small intestine reach the colon, in which absorption of water takes place, further solidifying stools. The proximal part of the gastrointestinal (GI) tract is loosely inhabited by microbes because of low pH, the presence of toxic bile acids, and high oxygen content (2). The GI tract gradually becomes more densely colonized by microbes distally. Depletion of dietary nutrients, such as fatty acids and carbohydrates in the intestinal lumen during the transit of food across the GI tract, renders the growth of many gut commensals dependent on nondietary host-derived energy sources by deconjugation of primary bile acids or degradation of mucin-derived glycans (3–7).

### Dietary impacts on the microbiome.

The gut microbiome is strongly influenced by the composition (8–10), amount, and timing (11–18) of its host’s diet. Mounting evidence suggests that the timing of feeding has a predominant effect on downstream metabolic and immune functions in microbiome-dependent and -independent manners. In a given person, substantial variability was noticed when identical meals were consumed at different times of the day (19). The intestinal microbiome exhibits diurnal oscillations that are driven by feeding patterns (15, 17, 18). Circadian-clock perturbation (commonly termed “jet lag”) induces a dysbiosis that is associated with glucose intolerance and obesity that are transferable by fecal microbial transfer (FMT) (15, 18). The transcriptomic landscape of nonintestinal organs was shown to oscillate as a function of feeding timing, which is (at least partially) regulated by corresponding oscillations in gut-derived serum metabolites (14, 18). An irregular feeding pattern may result in impairments of fundamental physiological functions, such as hepatic detoxification. The peculiar propensity of the gut microbiome to adapt to dietary perturbation is mirrored by the speed at which this adaptation takes place (20–24). Dietary constituents may support or impede the growth of particular microbes and also contain foodborne microbes, directly contributing to the net composition of the microbial genetic pool in the gut (9). Other dietary elements act as “immunomodulators” and can indirectly affect the microbiome composition in an immune-dependent manner via regulation of cellular and secreted immune effectors (25–28).

### Microbiome impacts on digestions and absorption.

**FIG 1:**
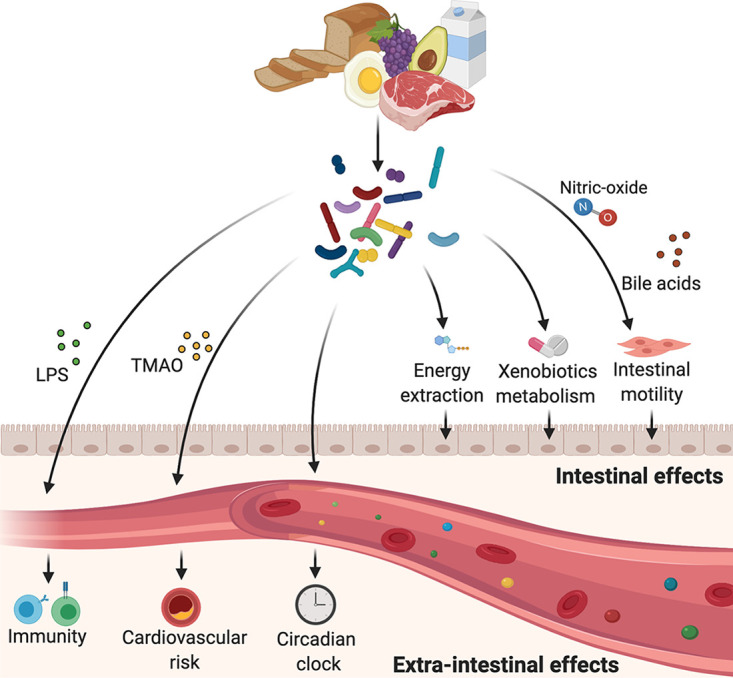
Examples of dietary microbiome cross talk. During digestion, food is decomposed to fat, proteins, carbohydrates, minerals, and other substances. Interactions between dietary habits and the intestinal microbiome result in alterations of various aspects of mammalian physiology in intestinal and nonintestinal organs. The image was created at BioRender. LPS, lipopolysaccharides; TMAO, trimethylamine *N*-oxide.

Host-microbiome metabolic interactions are bidirectional. Intestinal motility is regulated by bacterial metabolism of bile acids and bacterially induced nitric oxide production in a diet-dependent manner (Fig. 1) (29–32). Food choices are hypothesized by some to be subjected to microbiome influences, although evidence supporting that notion remains scarce (33–35). Postdieting weight recidivism, a common complication in nutritional clinical practice, is modulated by a persistent diet-altered microbiome “memory” (36), at least in rodent models. Adiposity and weight gain in various mammals can be modified with microbiome manipulation and were hypothesized to be governed by the gut microbiomes’ capacity to extract energy from diet (37–40). Promotion of weight gain is commonly achieved in livestock by antibiotic treatment (41, 42), a practice that is futile in germfree (GF) poultry (43), suggesting that microbiome manipulation by antibiotics may enhance dietary energy extraction. In healthy individuals, a short course of oral vancomycin (an antibiotic agent that is not absorbed systemically and therefore affects only the gut) attenuated dietary energy harvest compared with that after administration of a placebo, as mirrored by stool calorie loss (44). Interestingly, GF mice were previously reported to be resilient to deleterious effects of high-fat diet (HFD) feeding, such as weight gain and glucose intolerance (45, 46); however, findings from recent studies using various types of HFDs suggest that vivarium-dependent factors may differentially induce this trait (47–52).

### Major macronutrients and the microbiome.

Dietary fibers, also termed “glycans” or “polysaccharides,” are mainly plant-derived complex polymers of covalently linked simple carbohydrates (5). Humans are virtually devoid of enzymes that can decompose fibers, whereas gut bacteria express thousands of genes that encode carbohydrate-degrading enzymes (53). The products of primary and secondary fiber degradation are utilized by other members of the gut microbiome and the host in a convoluted web of cross-feeding (5, 6, 54, 55). A combination of person-specific fiber degradation capacity and the given fiber most probably determines the effects of fiber on host metabolism and the microbiome; however, in general, an increased intake of dietary fibers is associated with a higher overall microbial diversity, dominated by enrichment of *Bacteroidetes* and *Prevotella* spp. (20, 21, 56–58), and coupled to favorable metabolic and immunologic effects, such as improved insulin resistance and lower susceptibility to infection and malignancy (59, 60). Inversely, fiber deprivation leads to decreased microbial diversity, lower colonic bacterial butyrate production, barrier dysfunction, and susceptibility to perturbation and infection (21, 61–67). There is a notable interpersonal variability in complex and simple-carbohydrate digestion that is believed to be microbiome mediated and has been the subject of extensive research (as described in the next section) (59, 68–70).

Carbohydrates, proteins, and fats have all been shown to interact with the gut microbiome. Western diets that are rich in fat induce weight gain and insulin resistance by impairing intestinal barrier function and propagating a Toll-like receptor 4-mediated inflammatory response that is termed “metabolic endotoxemia” (51, 71–81). The resulting deleterious effects can be diminished by treatment with antibiotics (82) or phenocopied to another host by fecal microbial transfer (37). Proteins are metabolized by gut microbes into small metabolites, such as short-chain fatty acids (SCFAs), neurotransmitters, and organic acids, that have physiological effects both locally and systemically (83–87). A plethora of widely consumed dietary and nondietary constituents, such as emulsifiers (88–90), nonnutritive sweeteners (91–96), trehalose (97), probiotics (98–101), omega-3 fatty acids (102), and medications (103–105), were shown to feature considerable microbiome-mediated health impacts and are a subject of intensive research that is beyond the scope of this review.

### The microbiome as a “signaling hub.”

The intestinal microbiome generates downstream systemic signals, many of which are diet derived (106). One prominent example is the ketogenic diet, which aims at biochemically replacing carbohydrates with fat as a primary energy source through consumption of a low-carbohydrate, high-fat diet. This diet is commonly used in clinical practice to reduce seizure frequency in the treatment of drug-resistant epilepsy and is known to induce considerable microbiome and immune alterations; however, its mechanism of action remains unknown (107, 108). A recent study demonstrated that the ketogenic diet lacks an antiseizure effect in microbiome-depleted mice (either GF or antibiotic-treated mice) and that a fecal microbiome transfer from mice fed a ketogenic diet into mice fed a control diet induced a seizure-protective effect (109). A reduced amino acid gamma-glutamylation capacity of the ketogenic diet-associated microbiome was shown to elevate the seizure threshold in that mouse model of epilepsy. Collectively, evidence in support of an intensive cross talk between the gut microbiome and host nutrition, which may impact a variety of physiological and pathophysiological traits, is accumulating.

## THE GUT MICROBIOME IN PRECISION NUTRITION

Dietary habits constitute a strong driver of interpersonal variance in the gut microbiome composition, and its influence prevails over that of genetics by most estimates (23, 110–112). One example of person-specific microbiome impact on dietary physiological responses to consumed food focused on artificial sweeteners, mainly saccharin, and demonstrated that glycemic responses to these seemingly inert food supplements were driven by variations in the human microbiome (95). Moreover, adverse glycemic responses to saccharin could be predicted using machine learning by utilizing microbiome data collected before sweetener exposure (95). Indeed, a longitudinal concurrent daily dietary log and stool metagenomic sequencing throughout 17 consecutive days for 34 healthy individuals recently revealed markedly person-specific diet-microbiome interactions (113). Whereas some aspects of optimal nutrition unanimously apply, most are person specific and may differ in a population based on genetic and environmental factors. Within the environmental component, the gut microbiome accounts for some variation in subject-specific responses to diets, as do the timing of meals, time between meals, level of physical activity, and multiple other individualized features.

Adherence to dietary recommendations in the long term is a salient obstacle to dietary interventions (114, 115). A tailored intervention can potentially increase compliance, improve patient selection, and prevent weight gain-weight loss cycles that may predispose to adverse cardiometabolic health outcomes (116). Microbiome-based predictions of person-specific responses to some foods were demonstrated to be accurate and clinically beneficial in several studies. The baseline microbiome predicted the response to caloric restriction in mice. Interestingly, cohousing mice before dietary intervention resulted in a convergence of their microbiome configuration and a subsequent similar response to the dietary intervention (117). Nonnutritive sweetener consumption induces glucose intolerance, which is transferable to germfree mice by fecal microbial transfer and can be abrogated by antibiotics, suggesting a microbiome-dependent effect (95). Intriguingly, a subject-specific response to nonnutritive sweeteners was exhibited in humans, with the microbiomes of responders and nonresponders clustering separately (95, 118). Similarly, the glycemic response to different types of bread could be reliably predicted based on microbiome features (119). In contrast, 16S rRNA sequencing of stool microbiomes before the commencement of low-carbohydrate/fat diets was not predictive of weight loss success (120). Several studies on dietary interventions to treat obesity and metabolic syndrome have reported various associations between microbiome parameters and treatment efficacy. However, their heterogeneous design, small sample sizes, and short-term intervention profoundly limit their translational potential (111, 121–124). The same applies to a few studies assessing the low FODMAP diet (a diet low in fermentable carbohydrates) in the treatment of irritable bowel syndrome (IBS) (125–129).

Trimethylamine *N*-oxide (TMAO) is produced by intestinal microbes from dietary choline, which originates mainly in red meat. High TMAO levels are associated with adverse cardiovascular outcomes due to atherosclerosis and thrombosis (130–135). TMAO production is largely microbiome dependent and can be suppressed by antibiotics (133) or inhibition of bacterial enzymes (136). Considerable interindividual variability in TMAO production capacity exists across populations, with carnivores and vegans/vegetarians having on average higher and lower TMAO production capacities, respectively (131). Identification of individuals with a nonfavorable TMAO production capacity can serve as a source of microbiome-based personal nutrition recommendation and can be achieved without expensive sequencing by an oral carnitine challenge test (137). Unfortunately, such personalized predictions are not provided by nutritionists at this time, and recommendations to avoid red meat are generally dispensed to patients with high cardiovascular risk.

Dietary fibers are nutritionally beneficial, and their metabolism is almost entirely dependent on the expression of specific bacterial genes, potentially making them a focus of precision nutrition (59, 69, 138). Dietary guidelines recommend consumption of ∼30 g fiber a day for adults (or 14 g for every 1,000 cal), but such general recommendations are suboptimal due to several considerations (139). The chemical structures of molecules jointly referred to as fibers vary, and so do the identities and functions of the bacterial strains that can degrade them. Therefore, the effect that fibers may have on host health and the host’s intestinal microbial community is highly individualized (5, 54, 140). Hence, high interpersonal variability in metabolic outcomes and microbiome readouts is exhibited in clinical trials testing fiber supplementation (141–143).

Although the gut microbiome is a key determinant of a person’s response to fiber consumption, no reliable means of predicting a person-specific response to fiber supplementation exist to date, although some associations between clinical outcomes and microbiome features (community diversity and certain abundances of taxa, mainly the *Bacteroides*, *Prevotella*, *Bifidobacterium*, and *Ruminococcus* genera) have been suggested by multiple studies (21, 58, 59, 123, 141, 144–152). Habitual dietary fiber consumption may best predict the response to fiber supplementation more than any other microbiome parameter, and long-term multigenerational fiber deprivation leads to the extinction of fiber-degrading taxa, resulting in a hampered recovery of those taxa upon reintroduction of fiber (10, 153, 154). Considering the benefits of fiber consumption and the fact that fiber degradation is exclusively bacterial and highly variable, microbiome-driven prediction of person-specific fiber degradation capacity constitutes an exciting future challenge in clinical nutrition.

**FIG 2:**
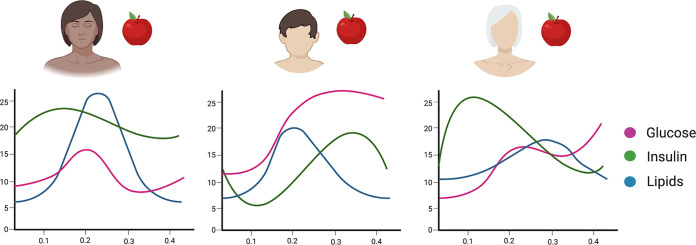
Person-specific postprandial responses. Genetic and nongenetic factors, such as age, the nature of a meal, habitual diet, level of physical activity, and the microbiome, account for considerable interindividual variability in energetic and endocrine postprandial responses, resulting in large differences in metabolic parameters following identical meals. The image was created at BioRender.

As previously discussed, gut microbes actively take part in carbohydrate metabolism and glucose homeostasis by degrading carbohydrates and by producing secondary bile acids and SCFAs that stimulate secretion of glucoregulatory hormones (e.g., glucagon-like peptide 1 [GLP-1], peptide YY [PYY]) (155–160). The postprandial surges in blood glucose levels (i.e., postprandial glycemic response, or PPGR) considerably vary between individuals, even following the ingestion of the same type and quantity of carbohydrates in identical meals or following exercise (Fig. 2) (119, 157, 161, 162). The Personalized Nutrition Project (PNP) demonstrated that a person-specific PPGR to real-life meals can be accurately predicted based on basic clinical parameters and microbiome data (163). The accuracy of the machine-learning pipeline that based its prediction on continuous glucose monitoring (CGM) data, stool microbiome sequencing, dietary logs, and other clinical variables from 800 individuals was validated in an additional validation cohort of 100 subjects. The algorithm predicted individual PPGRs better than models based on caloric/carbohydrate content only, and microbiome features accounted for the explained variability in PPGRs to various degrees. A personally tailored dietary intervention based on the algorithm’s predictions improved glycemic parameters in 26 prediabetic individuals. While some microbiome-based classifiers that were developed in a given geographical context exhibited poor accuracy when applied to subjects from different geographical origins (164, 165), the personalized nutrition concept was subsequently validated in another cohort of 327 subjects from a different geographical area (166). The clinical efficacy of a person-tailored dietary intervention based on the algorithm’s predictions in improving glycemic control in prediabetic individuals is currently being tested in a long-term randomized controlled trial (clinical trial NCT03222791).

The recent PREDICT1 study assessed subject-specific postprandial metabolic responses (19). Unlike the PNP, PREDICT1 also predicted postprandial triglyceride (TG) levels and insulin responses in addition to glucose. Furthermore, it included 230 twin pairs with genomic data that allowed the investigator to estimate the contribution of inheritance to postprandial metabolic responses. As with the PNP, clinical and metabolic parameters as well as microbiome and CGM data were collected from 1,002 healthy individuals (and from an additional 100 individuals in the validation cohort). Genetics accounted for 48%, 9%, and 0% of the variability in postprandial glucose, insulin, and triglycerides, respectively, whereas the stool microbiome accounted for only 6.4%, 5.8%, and 7.5% of postprandial variability in blood glucose, insulin, and TG, respectively. A meal’s macronutrient composition and timing in relation to previous meal/sleep/exercise are well-established effectors of PPGR and were also shown in the PREDICT1 study to surpass the microbiome in their PPGR predictive power. Notably, the predictive algorithm developed in the PREDICT1 study reached an accuracy in PPGR prediction similar to that of the PNP (Pearson’s correlation coefficients [*r*] were 0.7 and 0.77 between predicted and measured PPGRs, respectively) despite the different inputs and machine-learning approaches used. Prediction of postprandial TG and insulin in the PREDICT1 study were less accurate. In summary, both the PNP and PREDICT1 studies provide good-quality evidence that dietary recommendations can be optimized to be patient tailored.

## CURRENT CHALLENGES IN PRECISION DIETS AND FUTURE PROSPECTS

With these major advances in understanding the contribution of the microbiome to precision nutrition notwithstanding, many challenges need to be addressed in order to increase our mechanistic understanding of the forces shaping individualized human responses to food and the role that the microbiome plays in this complex and poorly understood process.

CGM systems are extremely pragmatic research tools, as they enable affordable real-life assessment of glucose levels in an outpatient setting without the inconvenience of a finger prick. However, the accuracy of CGM systems may pose a challenge in the nondiabetic setting. In a study funded by Abbott, a manufacturer of CGM systems, the concordance of CGM with direct capillary blood glucose measurement was <90% (167). Moreover, the within-individual variability in nondiabetics upon simultaneous PPGR measurement by two identical (19) or two different (168) sensor systems was not negligible. These differences may possibly stem from variations between sensors or from true differences in glucose kinetics in different anatomical locations.

While machine learning provides valuable insights into features possibly contributing to these physiological outcomes, their mechanistic elucidation merits further molecular-level research. Equally elusive are the potential roles of the viral, fungal, and parasitic microbiomes in contributing to personalized human responses to food, as well as roles played by niche-specific microbiomes along the oral and gastrointestinal regions. Additionally, better annotations of microbial reads currently constituting “dark matter” may enable us to refine and improve the utility of the microbiome, when coupled with other clinical features, in predicting food-induced human responses. Finally, as nutrition is estimated to impact a plethora of infectious, inflammatory, neoplastic, and even neurodegenerative processes, understanding of the causative food-induced and microbiome-modulated effects induced in the human host under these contexts may enable us to rationally harness precision nutrition as part of the therapeutic arsenal in these common and often devastating human diseases.
